# Bone-Breaking Bite Force of *Basilosaurus isis* (Mammalia, Cetacea) from the Late Eocene of Egypt Estimated by Finite Element Analysis

**DOI:** 10.1371/journal.pone.0118380

**Published:** 2015-02-25

**Authors:** Eric Snively, Julia M. Fahlke, Robert C. Welsh

**Affiliations:** 1 Department of Biology, University of Wisconsin–La Crosse, 1725 State Street, La Crosse, Wisconsin, United States of America; 2 Museum für Naturkunde, Leibniz-Institut für Evolutions- und Biodiversitätsforschung, Invalidenstraße 43, D-10115 Berlin, Germany; 3 Department of Radiology, University of Michigan, Ann Arbor, Michigan, United States of America; Duke University School of Medicine, UNITED STATES

## Abstract

Bite marks suggest that the late Eocence archaeocete whale *Basilosaurus isis* (Birket Qarun Formation, Egypt) fed upon juveniles of the contemporary basilosaurid *Dorudon atrox*. Finite element analysis (FEA) of a nearly complete adult cranium of *B*. *isis* enables estimates of its bite force and tests the animal’s capabilities for crushing bone. Two loadcases reflect different biting scenarios: 1) an intitial closing phase, with all adductors active and a full condylar reaction force; and 2) a shearing phase, with the posterior temporalis active and minimized condylar force. The latter is considered probable when the jaws were nearly closed because the preserved jaws do not articulate as the molariform teeth come into occulusion. Reaction forces with all muscles active indicate that *B*. *isis* maintained relatively greater bite force anteriorly than seen in large crocodilians, and exerted a maximum bite force of at least 16,400 N at its upper P^3^. Under the shearing scenario with minimized condylar forces, tooth reaction forces could exceed 20,000 N despite lower magnitudes of muscle force. These bite forces at the teeth are consistent with bone indentations on *Dorudon* crania, reatract-and-shear hypotheses of *Basilosaurus* bite function, and seizure of prey by anterior teeth as proposed for other archaeocetes. The whale’s bite forces match those estimated for pliosaurus when skull lengths are equalized, suggesting similar tradeoffs of bite function and hydrodynamics. Reaction forces in *B*. *isis* were lower than maxima estimated for large crocodylians and carnivorous dinosaurs. However, comparison of force estimates from FEA and regression data indicate that *B*. *isis* exerted the largest bite forces yet estimated for any mammal, and greater force than expected from its skull width. Cephalic feeding biomechanics of *Basilosaurus isis* are thus consistent with habitual predation.

## Introduction

### Cetacean Evolution

Modern cetaceans (Odontoceti and Mysticeti) emerged from archaeocete whales in the latest Eocene or earliest Oligocene, ca. 34 m.y.a. [1], [2], [3], [4]. Archaeocetes originated from terrestrial artiodactyls around the Paleocene-Eocene boundary, ca. 54 m.y.a. [3], [5], [6], with the earliest representatives of archaeocete whales appearing in the early Eocene [4].

The transition from life on land to life in the sea took place within archaeocetes throughout the Eocene, as is documented by various semiaquatic (protocetids, ambulocetids, and remingtonocetids) and fully-aquatic forms (basilosaurids) in the middle and late Eocene, respectively (for reviews see, e.g., [3], [4]). This transition brought about morphological and functional changes that affected not only the locomotor apparatus, sensory and reproductive organs, but also feeding and diet [3], [7], [8], [9], [10], [11], [12], [13]. Isotopic and morphological studies [14], [15], [16] show that the transition to a marine environment happened relatively fast, and that semiaquatic forms were likely already marine.

### Feeding and Diet

Primitive terrestrial artiodactyls had bunodont teeth, and were most likely herbivorous and chewed their food [13]. Modern whales, on the other hand, do not masticate. Mysticetes filter-feed, while odontocetes capture their prey and swallow it whole or in large pieces. Suction feeding is also widespread in both groups [17], [18], [19].

Shearing facets on the cheek teeth of archaeocete whales indicate that archaeocetes chewed their food. Pakicetids and protocetids had a protocone on their molars, indicating that some grinding function was retained. The cheek teeth of basilosaurids were mediolaterally compressed and lacked grinding surfaces [13], [20]. Fahlke et al. [21] observed tooth wear and bite marks suggesting that the basilosaurid *Basilosaurus isis* used a single, orthal-retractional occlusal movement to puncture, crush, and shear its food.

Stomach contents of the basilosaurids *Basilosaurus cetoides* and *Dorudon atrox* consist of different teleost fish and, in the case of *B*. *cetoides*, sharks of up to 50 cm in length [10], [22]. Microwear analysis suggests that archaeocetes generally had quite a mixed diet including crustaceans and mollusks besides fish. In some species, e.g., *Basilosaurus isis*, tooth wear indicates the consumption of large hard objects such as vertebrate bones [23]. Very destructive tooth wear in *B*.*isis* and the protocetid *Babiacetus* has been interpreted as evidence of forceful crushing of large, hard objects, such as mammal bones, thus indicating the consumption of meat [13], [21]. Fahlke [24] matched morphology and positions of bite marks on skulls of juvenile *D*. *atrox* to the dentition of an adult *Basilosaurus isis*, suggesting that *B*. *isis*, like the modern killer whale (*Orcinus orca*), included other cetaceans in its diet.

### Estimating bite force in *Basilosaurus isis*

Some carnivorous vertebrates exert high bite forces to comminute bone, and bite marks and tooth wear [21], [23], [24] strongly suggest that *Basilosaurus isis* applied such forces on its prey. Estimating bite forces of *B*. *isis* enables us to test its attribution as the animal that left bite traces on juvenile *Dorudon*, to determine relative forces at different teeth, and hence to infer aspects of its feeding behavior. Many authors have estimated bite force by using Thomason’s [25] dry skull method to approximate muscle force [26], and Finite Element Analysis (FEA) to obtain reaction forces at the teeth and jaw joint. In mammals and other synapsids, including *B*. *isis*, the zygomatic arches and braincase delimit anatomical cross-sectional areas (ACSA) of jaw muscles [25], [27]. Multiplying ACSA by a specific tension (force/area) gives an estimate of adductor muscle force. This initial estimate can be corrected for muscle pennation angles [25], other aspects of muscle function [28], and refined specific tensions [29], and checked against experimental results for living animals [30], [31]. Muscle forces are then applied to FEA models, which are constrained at the jaw joint and bite points to obtain food and joint reaction forces.

Modelling *Bailosaurus* bite force with tooth constraints alone can assess the effectiveness of hypothesized orthal retractional shear on the food, as the teeth near occlusion. Bite force modelling with FEA usually assumes maximal force with all adductors active, and the upper and lower jaws in full articulation. With a retractive, food-shearing component to jaw closure evident from *Basilosaurus* bite marks [21], joint reaction forces would be minimized [32], [33], [34] and food reaction forces maximized. In such a bite scenario, the mandible would behave temporarily as a direct link between the cranium and the food, rather than a lever actuated about centers of rotation at the jaw joints[32], [33], [34]. Physical manipulation of the original *B*. *isis* mandible relative to the cranium confirms that the articular condyle of *B*. *isis* shifts anteriorly out of the cotyle as large molariform teeth occlude; these kinematics and morphology are currently under study for more extensive treatment. The animal’s application of tooth reaction forces hence would be more versatile than in carnivorous animals with tightly articulating jaws, such as felids, mustelids, and crocodylians. FEA enables virtual activation of only those adductor muscles that would cause the hypothesized shearing orthal retraction of the lower jaw.

We combine the dry-skull method with FEA to model and estimate bite force for *Basilosaurus isis* as a primarily vertical, crushing bite with all adductor muscles activated, and with a shearing load case powered by the posterior temporalis alone. Because *B*. *isis* tooth wear and bite marks on *Dorudon atrox* indicate preferential bite positions, it is possible to localize estimates of bite force to functionally critical locations. Such bites by *B*. *isis* would be analogous to those alligators exert with their molariform teeth to break turtle shells [30], and jaguars biting with their canines into the crania of peccaries [35]. Bite force estimates for *B*. *isis* can facilitate comparisons of function and absolute bite force between predatory aquatic tetrapods with skulls in the 0.8–2 meter range, including the pliosaurs *Kronosaurus* and *Pliosaurus* [36], [37], large crocodilians [30], [36], [38], and more recent cetaceans. These analyses of *Basilosaurus* can anchor future comparative evolutionary studies of bite force in archaeocetes during their land-sea transition, and of bite force in more derived whales [39].

**Fig 1:**
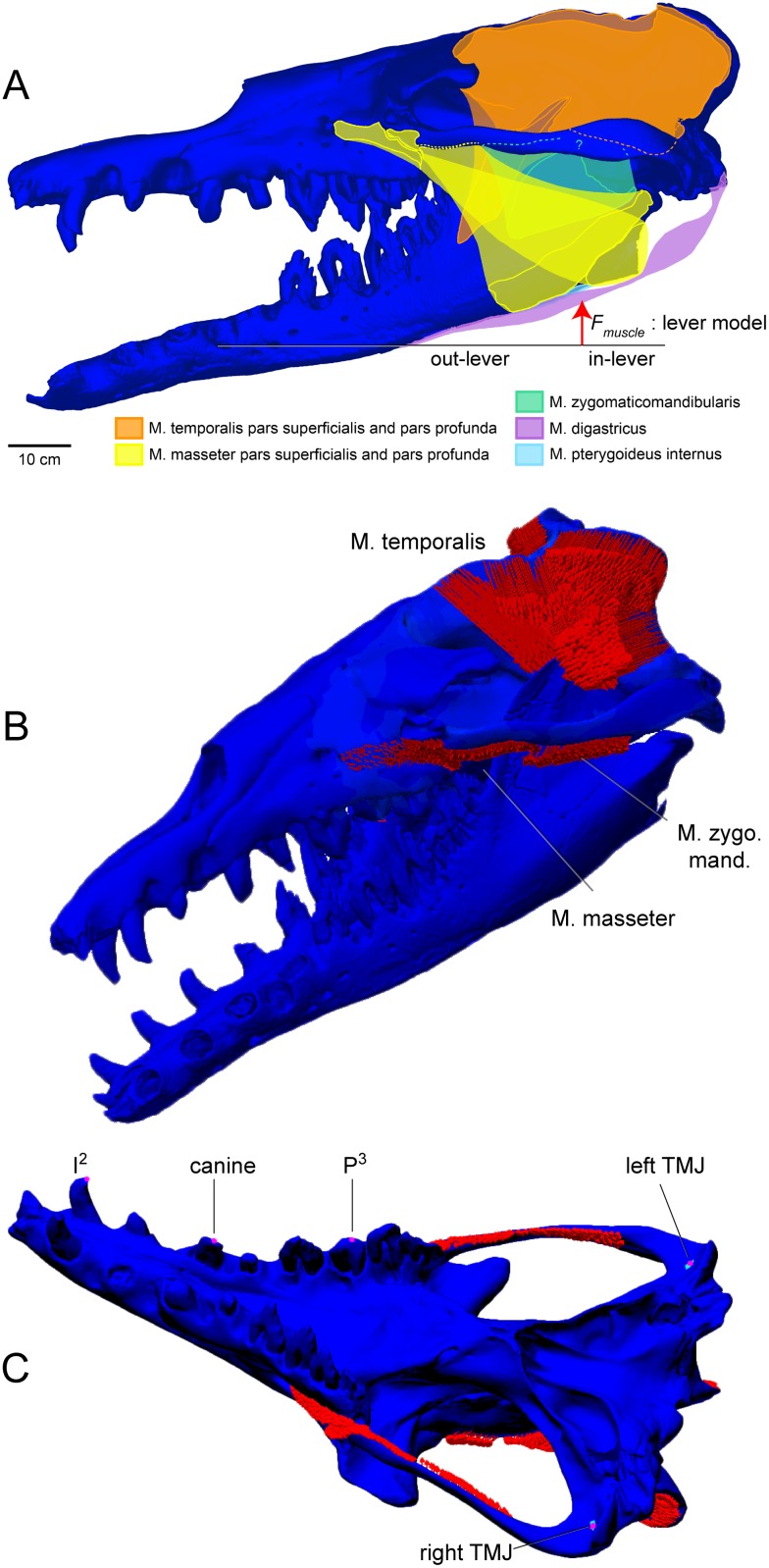
Jaw muscles of *Basilosaurus isis* and constraints for FEA. A. Jaw muscles of *B*. *isis* and muscle vector and moment arms for leverage-based force calculation. B. Adductors (red arrows) mapped onto a CT-based finite element model of the cranium of *Basilosaurus isis* WH-74, including the dentaries. C. Constraints for finite element analyses. In the all-muscles active analysis, the cranium was fully constrained at the left and right temporomandibular joints (TMJ), and tooth constraints were applied in respective bite analyses. In the posterior shear analysis, the only active muscles are the posterior temporalis, and only P^3^ is constrained.

With its enormous skull and body size, *Basilosaurus* is an outlier among carnivorous mammals for which bite force data are available. Thus, we compare our modeled bite force with estimates for other mammals [26], [29] to test whether *B*. *isis* had a particularly forceful bite for a mammal with its skull dimensions. Previous bite force estimates are based on diverse methods, including the dry skull method [25] and FEA, and comparisons among these modeled estimates warrant caution. Notably, dry skull mammal studies present a comprehensive and rigorous database of bite force estimates, and these estimates are only slightly lower than FEA results, which have muscle forces distributed across attachments 27], [36], [37] rather than between estimated centers of pull [25], [26], [29]. The current study’s bite force comparisons between *B*. *isis* and other mammals will therefore be worthwhile and testable by applying FEA [28], [30], [31] to more carnivorous mammals in Wroe and colleagues’ original results [26]. We construct a simple lever model of *B*. *isis* (Fig. 1) to check correspondence between simplified dry skull and 3D FEA approaches.

### FEA approach: testing bite force with plate element and solid models from surface scans

Normally in FEA of vertebrate skulls, a virtual surface derived from CT data is meshed volumetrically, creating a solid mesh of internal brick elements. Although such a volumetric FE mesh is necessary to resolve internal stresses, simpler models can be used to test hypotheses of bite force and other aspects of structural behavior [31], [40], [41], [42], [43], [44]. We introduce two surface-based models of *Basilosaurus isis*, derived from a CT scan of a cast reconstructed from separate but perfectly articulating original bones, to explore the precision of different FE models for obtaining bite reaction forces. The original fossils could not be safely assembled and scanned together, and the skull’s internal structure is currently being described. However, our bite force estimates based on surface data can be tested in the future against models that include internal CT data of individual bones.

To estimate bite force in *Basilosaurus isis*, our FE models respectively use thickened plate elements [45] and a cavity-filling solid mesh to approximate 3D cranial geometry, bracketing the complex internal anatomy (with braincase and sinus cavities) with hollow and fully solid structures. For structures consisting mostly of discrete walls (such as a vertebrate cranium) whose width is 10% or less than the whole object’s dimensions, plate elements have advantages over a solid internal mesh of those walls. Plate elements are computationally time-efficient, enabling large meshes, and testing with many loadcases and resolutions to estimate peak stresses (see S1 Text). Because plate elements can vary in thickness, they enable us to explore possible stress and strain distribution in hollow structures modeled with external surface scans from fossil CT scans with suboptimal internal resolution, and of skulls reconstructed from CT scans of individual bones as in the current study.

Element type and thickness of plate elements theoretically have little effect on interpretations of theoretical feeding forces if the cranium deforms little. To compare the effects of a solid mesh versus plate (and modeled bone) thickness on resulting reaction forces, we ran a sensitivity analysis by varying model type—a solid volume and three different plate thicknesses—with muscle forces held constant. We predicted that stress and strain values would decrease linearly with increasing plate thickness, but that theoretical food and jaw joint reaction forces would remain consistent regardless of plate thickness.

## Materials and Methods

### Specimen and Manual Preparation

WH-74 (WH for Wadi Al-Hitan) is a virtually complete skeleton of an adult individual of *Basilosaurus isis* with a total body length of ca. 16 m. It was excavated in the shallow-marine deposits of the late Eocene (Priabonian) Birket Qarun Formation of Wadi Al-Hitan, Fayum Province, Egypt (ca. 150 km southwest of Cairo), in 1989 and 2005. For more geographical, geological, and paleoenvironmental information on Wadi Al-Hitan, consult Gingerich [46] and Peters et al. [47].

WH-74 is currently housed in trust at the University of Michigan Museum of Paleontology (UMMP), Ann Arbor, Michigan, USA. The cranial elements and dentaries were found disarticulated in the field. The individual cranial elements included the left premaxilla and maxilla (disarticulated), the right premaxillae and maxilla (articulated), the frontal shield (frontals, parietals, and nasal bones in articulation), the articulated braincase/basicranium, both isolated squamosals, and left and right bullae. No taphonomic deformation was noted except for a slight mediolateral flattening of the right dentary. After preparation, the individual cranial elements of WH-74 were molded and casted, and the casts reassembled into a whole skull at the UMMP. Our skull length estimate for the entire skull of WH-74 is 113 cm.

All elements of the reconstructed skull belong to WH-74, and no other individual was used for the digital reconstruction. At least one disarticulated jugal and both pterygoid bones were confirmed after our reconstruction, and confirmed the accuracy of the reconstructed skull and cast. The completeness of these remains, their individual taphonomic integrity and perfect re-articulation with each other, and similarity to other taphonomically intact *Basilosaurus* skulls engender confidence in the cast skull for biomechanical study.

### Digital Preparation

Cranial parts, dentaries, and the composite cast of the cranium of WH-74 were scanned using computed tomography (CT) at the University of Michigan Department of Radiology (scanner GE HD-750). In-plane resolution was 0.879 mm, and slice thickness was 0.625 mm. Three-dimensional (3D) surfaces were extracted from image stacks using Amira 5.0 and post-processed in Materialise Magics 14. Slight taphonomic flattening of the right dentary was removed in Autodesk 3ds Max 2010 with reference to the undistorted left dentary and other specimens by bending the surface manually to allow for best-fit occlusal articulation of the upper and lower tooth rows. The dentaries were aligned with the cranium using Materialise 3-Matic 4.4, following the best possible dental occlusion.

### FE model geometry and material properties

For the plate element models, the cranium and dentary surfaces were exported from Materialise Magics as. stl files (binary, little endian) into Autodesk Simulation. The complete model is available as S1 Dataset, under Supporting Information. Element type was set to plate elements. To assess the sensitivity of bite force and stress to plate thickness, four models were constructed with respective thicknesses of 0.5, 1, 1.5, and 2 cm, in the range found for posterior dentary bone of *Basilosaurus isis* [12]. Thicknesses of 1–2 cm ensured that the teeth and sagittal and nuchal crests would be solid, and that the braincase and airways would be hollow. However, CT scans reveal that the frontals and parietals are thicker than 2 cm between their external surfaces and the endocranial cavity.

For the solid mesh model, we imported the surface into Materialise Mimics for solid meshing in Nastran format (.nas). This model was a simplified representation of the cranium because all internal cavities were meshed solid. Autodesk Simulation did not accept the model, so we imported the mesh into Strand7 for solving. We subdivided the mesh in Strand7 to produce a model with 1.256 million four-node tetrahedral elements. All material properties, constraints, and muscle forces were applied in Strand7 exactly as for the plate models in Autodesk Simulation. Autodesk Simulation and Strand7 both use standard Nastran-related mathematical code in their FE solvers, and their results are precisely the same with identical models.

In the absence of data on material properties of archaeocete cranial tissues, we initially assigned isotropic properties to the entire model in an overlapping range for mammalian compact bone and dentine (elastic modulus *E* = 17.4 GPa, Poisson’s ratio = 0.34 [48]). The enamel on the teeth of *Basilosaurus* is very thin, so to preliminarily examine stresses in within the teeth we considered properties of dentine to be appropriate, especially where the enamel has worn down. Because whales are cetartiodactyls, we further ran an analysis using properties of bovid Haversian bone (*E* = 10 GPa, Poisson’s ratio = 0.4 [40]). The lower stiffness (stress/strain) of Haversian bone will give unrealistically high strain readings for the teeth, but may be more realistic for the cranial bone. Because material properties were considered isotropic (independent of direction), Autodesk Simulation and Strand7 estimated shear modulus from *E* and Poisson’s ratio.

### Muscle force estimates

In order to estimate the bite forces generated by *Basilosaurus isis* during the occlusal movement of the lower jaw, the position, magnitude and direction of pull of the elevator muscles of WH-74 were reconstructed in Autodesk Simulation. Muscles exert isometric force when velocity is 0 m/sec, as might occur when teeth of a biting animal encounter resistant food. This force will equal a cross-sectional area of the muscle times a specific tension *ST* (force/area). Isometric specific tension in vertebrates is often set at 30 N/cm^2^ [25], [49]. Complexities of muscle geometry, including pennation and varying fiber lengths, can increase this specific tension for a given anatomical cross sectional area (ACSA), by increasing the physiological cross sectional area (PCSA: [50]; dramatically in some reptiles: [51], [52]). We apply two specific tensions to *B*. *isis* simulations: 30 N/cm^2^ assuming simple geometry, and 37 N/cm^2^ to account for realistic pennation of mammalian jaw adductors [25], [29], [53].

Cross-sectional areas of the musculus (m.) temporalis were estimated with the dry-skull method [25]. A 3D,. stl surface model of the articulated *Basilosaurus isis* cranium and dentaries was exported from Materialize Magics into Autodesk Simulation. A posterodorsal-view screen capture of the model, with scale and without perspective distortion, imaged the area of m. temporalis between the braincase and zygomatic arches.

Anatomical cross-sectional areas (ACSA) were estimated using two methods. First, measurements of major and minor radii enabled approximation of the areas as ellipses, using the equation *ACSA = π × r*_*maj*_ *r*_*min*_. Second, the image was imported into ImageJ (National Institutes of Health of the United States: rsb.info.nih.gov/ij/), its scale set to the original fossil’s size, and ACSA calculated within anatomical regions traced manually with the pen tool.

Estimating ACSA for the masseter was more difficult than for the well-delineated temporalis. The zygomatic arches and origin areas for m. masseter are slender in *Basilosaurus isis*, and the masseteric fossa is shallow. As a starting point for forces of m. masseter, we assumed that its ACSA was 10% that of m. temporalis. This area is reasonable considering the length of the masseter’s origin, but may be an overestimate considering the large ACSA of the temporalis. Varying the area would multiply ad hoc assumptions with minimal realistic effects on overall bite force, and we suspect that the masseter assisted the medial pterygoid in laterally positioning the lower jaws (see below).

Origins of temporal and masseter muscles were positioned on the model cranium of WH-74 based on osteological correlates, i.e., recognizable attachment surfaces, on the cranium and dentaries. Vectors were distributed homogeneously to nodes of the FE model on these surfaces. Force directions were estimated by measuring distances from the centroids of muscle origination to their insertion surfaces on the dentaries (Table 1, Table 2), which were digitally aligned to the cranium with the mouth slightly opened. From these dimensions, x, y, and z force components were calculated trigonometrically. Muscle force magnitudes were divided equally among divisions of the temporalis and masseter. Origins for the temporalis include the nuchal crest, the temporal region anteroventral to the nuchal crest, and anterior, middle, and posterior regions of the sagittal crest. We found that attachment surfaces of the masseter group represent superior and inferior m. masseter, and m. zygomaticomandibularis (Fig. 1A), in contrast to Uhen’s [10] results for *Dorudon atrox*, but in agreement with the results of Carpenter and White [54] for another basilosaurid, *Zygorhiza kochii*. Magnitude for each temporalis division was therefore 1/5 of the overall magnitude calculated for the muscle, and for the masseter 1/3 of its full force magnitude was applied to each of its divisions.

**Table 1:** Inputs and results for estimating adductor muscle forces in *Basilosaurus isis*, assuming 30 N/cm^2^ baseline isometric specific tension (ST).

Left side	TemporalisMuscle Area (cm^2^)	Muscle Force 30 N/cm^2^ *F*_*temporalis*_						
	896	26889	Origin to insertion (mm)					
	**Division**	***F***_***division***_ **=** *F*_*temporalis*_/5	x	y	z	***Fx***	***Fy***	***Fz***
**m. temporalis**	temporal	5378	148	47	12	5105	1641	412
	nuchal	5378	171	73	-91	4446	1890	-2363
	sag. crest p	5378	63	166	-115	1607	4227	-2910
	sag. crest m	5378	-50	173	-126	-1221	4225	-3095
	sag. crest a	5378	-3	115	-136	-87	3472	-4106
**m. masseter**		***F***_***masseter***_						
		2689						
	**Division**	***F***_***division***_ **=** *F*_*masseter*_/3	X	y	z	***Fx***	***Fy***	***Fz***
	Superior	896	-399	13	-215	-789	26	-425
	Inferior	896	-181	5	-169	-655	17	-612
	zygomat.	896	49	-77	-123	286	-449	-721

Right side	Temporalis Muscle Area (cm^2^)	Muscle Force 30 N/cm^2^ *F*_*temporalis*_						
**m. temporalis**	760	22806	Origin to insertion (mm)					
	**Division**	***F***_***division***_ **=** *F*_*temporalis*_/5	X	y	z	***Fx***	***Fy***	***Fz***
	temporal	4561	156	-63	16	4207	-1709	433
	nuchal	4561	170	-113	-108	3359	-2239	-2123
	sag. crest p	4561	69	-185	-107	1406	-3755	-2174
	sag. crest m	4561	-61	-165	-109	-1335	-3644	-2397
	sag. crest a	4561	-6	-127	-147	-149	-2981	-3449
**m. masseter**		***F***_***masseter***_						
		2281						
	**Division**	***F***_***division***_ **=** *F*_*masseter*_/3	X	y	z	***Fx***	***Fy***	***Fz***
	superior	760	-423	-46	-207	-680	-74	-332
	inferior	760	-216	-3	-152	-621	-10	-438
	zygomat.	760	20	88	-79	128	559	-499

Areas are estimated in ImageJ, and multiplied by ST to calculate overall m. temporalis force *F*_*temporalis*_. Total m. masseter *F*_*masseter*_ were assumed to be 10% of temporalis forces. Forces applied in FEA to origination areas of these muscles were calculated by dividing their total force by the number of divisions (5 for m. temporalis, 3 for m. masseter). Distances from origin to insertion centroids of the muscle divisions were used to calculate their *F*_*xyz*_ directional components. Abbreviations: sag. crest p = sagittal crest posterior; sag. crest m = sagittal crest middle; sag. crest a = sagittal crest anterior; zygomat = m. zygomaticomandibularis.

**Table 2:** Adductor muscle forces in *Basilosaurus isis*.

Left side	Temporalis Muscle Area (cm^2^)	Muscle Force 37 N/cm^2^ *F*_*temporalis*_						
	896	33163	Origin to insertion (mm)					
	**Division**	***F***_***division***_ **=** *F*_*temporalis*_/5	X	y	z	***Fx***	***Fy***	***Fz***
**m. temporalis**	temporal	6633	148	47	12	6296	2024	508
	nuchal	6633	171	73	-91	5483	2331	-2914
	sag. crest p	6633	63	166	-115	1981	5214	-3590
	sag. crest m	6633	-50	173	-126	-1506	5211	-3817
	sag. crest a	6633	-3	115	-136	-108	4283	-5064
**m. masseter**		***F***_***masseter***_						
		3316						
	**Division**	***F***_***division***_ **=** *F*_*masseter*_/3	X	y	z	***Fx***	***Fy***	***Fz***
	superior	1105	-399	13	-215	-973	32	-524
	inferior	1105	-181	5	-169	-808	21	-754
	zygomat.	1105	49	-77	-123	353	-554	-889

Right side	Temporalis Muscle Area (cm^2^)	Muscle Force 37 N/cm^2^ *F*_*temporalis*_						
	760	28127	Origin to insertion (mm)					
	**Division**	***F***_***magnitude***_	X	y	z	***Fx***	***Fy***	***Fz***
**m. temporalis**	temporal	5625	156	-63	16	5188	-2108	534
	nuchal	5625	170	-113	-108	4143	-2761	-2619
	sag. crest p	5625	69	-185	-107	1734	-4631	-2681
	sag. crest m	5625	-61	-165	-109	-1647	-4494	-2956
	sag. crest a	5625	-6	-127	-147	-184	-3676	-4254
**m. masseter**		***F***_***masseter***_						
		2813						
	**Division**	***F***_***division***_ **=** *F*_*masseter*_/3	X	y	z	***Fx***	***Fy***	***Fz***
	superior	938	-423	-46	-207	-839	-91	-409
	inferior	938	-216	-3	-152	-766	-12	-540
	zygomat.	938	20	88	-79	158	689	-616

Inputs and results for estimating adductor muscle forces *F*_*realisitc ST*_ in *B*. *isis*, assuming 37 N/cm^2^ isometric specific tension (ST), a realistic value incorporating pennation. Areas are estimated in ImageJ, and multiplied by ST to calculate overall m. temporalis force *F*_*temporalis*_. Total m. masseter *F*_*masseter*_ were assumed to be 10% of temporalis forces. Forces applied in FEA to origination areas of these muscles were calculated by dividing their total force by the number of divisions (5 for m. temporalis, 3 for m. masseter). Abbreviations: sag. crest. p. = sagittal crest posterior; sag. crest. m. = sagittal crest middle; sag. crest. a. = sagittal crest anterior; zygomat = m. zygomaticotemporalis.

No forces were estimated for the pterygoid muscles, because their attachments are ambiguous and the function of these muscles in mammals is inconsistent with powerful adduction. The lateral pterygoid protracts and opens the lower jaw [55], and ensures proper position and function of the temporomandibular joint meniscus. Inclusion of the medial pterygoid (m. pterygoideus internus) would increase estimated adductor force, indicating an underestimate in our values. However, the pterygoid bones are partially broken and were reconstructed in the composite cast of WH-74, and no unambiguous muscle attachment surfaces could be identified on the original bones. Thus we could not confidently reconstruct the position and extent of the origin m. pterygoideus internus, although the muscle’s overall morphology probably resembled that of other mammals (Fig. 1A). Divisions of m. pterygoideus internus normally insert onto the posteromedial surface of the dentary, which is a thin flange of bone in *Basilosaurus isis* (Fig. 1C) without clear demarcations of muscle scars. These divisions are highly active and effective during lateral grinding in pigs [56], [57], [58]. All of these factors suggest low adductor force of any one division of m. pterygoideus internus, and little contribution to adduction force compared with the temporalis muscles.

### Load cases and constraints for bite force at P^3^

We applied two loading and constraint regimes to the modeled cranium of *Basilosaurus* to determine reaction force at P^3^, a tooth inferred strongly as indenting specimens of *Dorudon* crania [24].

1) One load assumed that all tested adductors were fully active. Constraints at P^3^ and the left and right articular condyles gave respective tooth and jaw reaction forces. This would more likely occur at relatively high gape angles, as the teeth would be in contact with a large food item. Although a crushing bite at this gape angle may not be realistic, it enables us to compare forces with those of large-headed reptiles that apply such bites, such as *Alligator* feeding on turtles.
2) Another load assumed orthal retractional occlusion, with only the posterior temporalis active acting to retract the jaws, and masseter active for slight adduction. Under these conditions, the only constraint was at P^3^ where it would meet the food. Force magnitude of the posterior temporalis was its proportion of the total adductor force.

### Regression and FEA for comparing bite forces of *Basilosaurus* and other carnivorous mammals

Using data from Wroe et al. [26] on carnivorous mammals (Table 3), we log-10 transformed basal skull length and skull width across the zygomatic arches (both in cm), and linearly regressed these quantities against log-10 of canine bite force (N [26]). Wroe et al. [26] used the dry skull method to estimate force for both extant and extinct mammalian carnivores, instead of estimating extinct forces using a modern-specimen regression. Our regressions thus avoid double-counting the influence of bite force in the extinct forms, a danger if their forces were estimated statistically. Regression equations gave us expected bite forces for *Basilosaurus isis* at the position of the canines in other carnivorous mammals (more anterior than the canine in *B*. *isis*). These regression-based force estimates are highly tentative, because the skull of *B*. *isis* is over three times longer and wider than the largest specimens in Wroe et al.’s [26] sample. As an additional check, we estimated bite force of *B*. *isis* with a lever model (Fig. 1), assuming (1) a vertical force through the centroid of ACSA, and (2) multiplying this by the sin of the angle between this vertical force and the sagittal crest, where most of the adductor muscles attach.

**Table 3:** Reaction forces and von Mises stresses with unilateral bites in *Basilosaurus isis*.

Loadcase 1: Full adduction (all adductors active)							
Plate thickness (mm)	*F*_*resultant*_ *P* ^*3*^ (N)	*F*_*Z*_ *P* ^*3*^ (N)	*σ*_*vM*_ *P* ^*3*^ (Mpa)	*F*_*resultant* JA LEFT_ (N)	σ_vM JA LEFT_ (MPa)	*F*_*resultant* JA RIGHT_ (N)	*σ*_*vM* JA RIGHT_ (MPa)
5	16453	-16128	399	9256	300	12854	386
10	16448	-16041	162	9233	94	12558	129
15	16458	-15975	94	9317	49	12400	65
20	16483	-15921	63	9384	33	12282	40
**Average *F***	**16461**	**-16016**		**9298**		**12523**	

**Loadcase 2: Orthal retraction (posterior temporalis and masseter active)**							
Plate Thickness (mm)	*F*_*resultant*_ *P* ^*3*^ (N)	*F*_*x*_ *P* ^*3*^ (N)	*σ*_*vM*_ *P* ^*3*^ (Mpa)	n/a	n/a	n/a	n/a
20	20427	19107	283				

Shown are reaction forces (N) and von Mises stresses (*σ*_*vM*_: MPa) with unilateral bites in *B*. *isis* at *P* ^*3*^ and the left and right jaw articulations (JA). Muscle specific tension is 37 N/cm^2^. Results for Loadcase 1 (with all tested adductors active) are for models with four different thicknesses of plate elements. In addition to resultant forces (*F*_*resultant*_), vertical reaction force (*F*_*Z*_) is reported at *P* ^*3*^. Results for Loadcase 2 (orthal retraction with only the posterior temporalis and masseter activated) include the x-axis component of the reaction force. The high magnitude of *F*_*X*_ indicates high shear force.

We compared regression estimates of *Basilosaurus isis* bite force with reaction forces from FE simulations. We used FEA to estimate forces at the canine and the caniniform I^2^ in *B*. *isis* (at a similar position to the canine in other mammals: [26]), by constraining these teeth in simulations of unilateral bites. In these analyses all muscle forces were active (as in P^3^ bite simulation 1), with the FE model set to 2 cm plate thickness, E = 17.4 GPa, and Poisson’s ratio = 0.34.

### Testing the precision of bite force results with different plate element models

We used FEA primarily to calculate bite forces. However, we also compared von Mises stresses between models of different plate thickness, to examine the sensitivity of bite reaction forces to both plate thickness and to stress magnitudes. Von Mises stresses represent the entire stress tensor as a scalar, enabling comparison of distortional stresses and risk of failure in ductile materials [48], such as bone under low strains. Von Mises stress is proportional to strain energy density, and values above yield or ultimate stress are good predictors of material and structural failure. We would expect higher stress (force/area) in thinner-plate models. If tooth reaction force is consistent regardless of plate element thickness and stress, we can conclude that plate element modelling is useful for estimating bite forces. Conversely, we can reject the current application of the plate FE method if tooth reaction forces vary greatly between thick and thin plate models, indicating high sensitivity to plate thickness and von Mises stress.

## Results

### Muscle and reaction forces

Anatomical CSA and inferred muscle forces of the temporal and masseter muscles are asymmetrical between the left and right sides for *Basilosaurus isis* (Table 1, Table 2). Areas measured in ImageJ are 896 cm^2^ on the left side and 760 cm^2^ on the right, for respective m. temporalis force magnitudes of 33,163 N and 28,127 N, assuming 37 N/cm^2^. This discrepancy is unsurprising, considering the asymmetry of *Basilosaurus* and other whales’ skulls [12]. Total adductor forces at this specific tension are 61,291 N (Table 2). Anatomical CSA and forces based on ellipse dimensions were 67% of values from ImageJ, suggesting that areas approximated as ellipses will underestimate forces.

**Fig 2:**
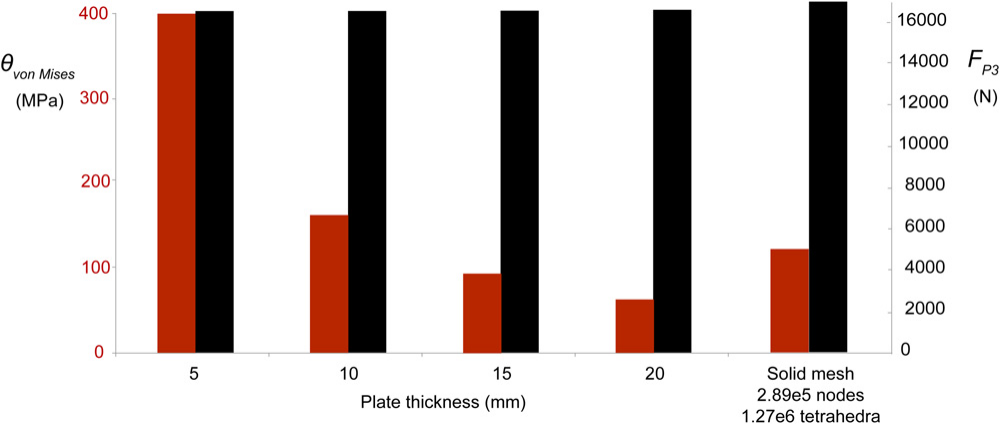
Sensitivity of P^3^ von Mises stresses and reaction forces to plate element thickness and a solid volume mesh. Note that bite force estimates at the third premolar (*F*_*P3*,_ black bars, right scale) are consistent (16,453 N-16,483 N) regardless of plate element thickness. By contrast, peak von Mises stresses (red bars, left scale) vary widely with varying plate thickness, from 399 MPa at 5 mm plate thickness to only 160 MPa at 10 mm thickness, and 64 MPa at a realistic 20 mm. These results indicate that plate element FEA is precise and useful for estimating bite forces, but not recommended for estimating stress magnitudes unless element thickness matches that of the original structure. *F*_*P3*_ for the volume mesh is 16,541 N and peak stress is 51 Pa.

**Fig 3:**
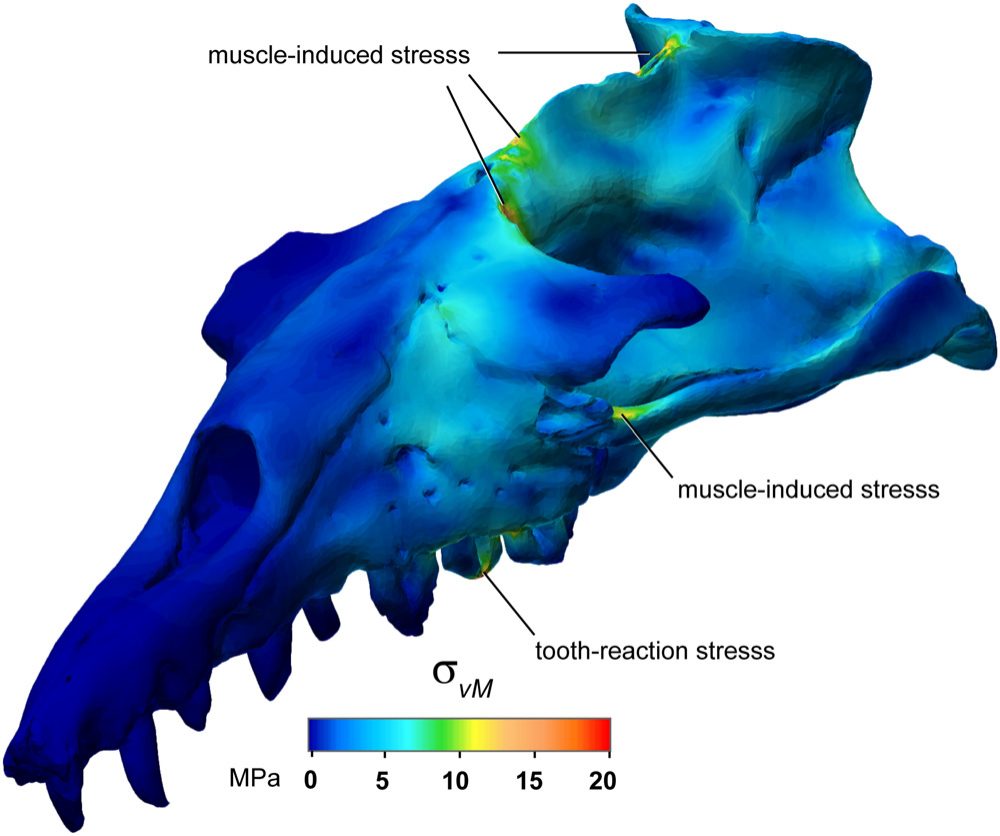
Exploratory plate-model distribution of von Mises stresses. Von Mises stresses (σ_vM_; maximum shown here of 20 MPa) in *Basilosaurus isis* biting on its upper third premolar, assuming muscle specific tension of 37 N/cm^2^. Oblique view. Note an arc of evident stress in the maxilla dorsal to the bite point, and muscle-induced stresses from the force of m. temporalis.

Under the load case with full muscle activation and constraints at P^3^ and both jaw joints, all reaction forces scaled linearly with specific tension. With the realistic *ST* of 37 N/cm^2^ [29], the average theoretical food reaction force at P^3^ was 16,461 N (Table 3). The joint reaction force was greater on the right side at 12,523 N, versus 9,298 N on the left, despite greater muscle force and a bite point both on the left side (Table 3). Reaction forces were insensitive to element thickness or type and hence to von Mises stress (Fig. 2), with a magnitude at P^3^ of 16,483 N for elements of 2 cm thickness, only 0.18% greater than 16,453 N for 0.5 cm thickness (Fig. 2). Vertical (z-axis) bite reaction force was more variable, with relative forces reversed at 1.28% greater for the model at 0.5 cm thickness than at 2 cm. Stress magnitudes (Fig. 2) do not affect hypothetical distributions of stress (Fig. 3) in tests with models of different plate thickness.

Under the load case with only the posterior temporalis and masseter active and constrained only at P^3^, reaction force magnitude at the left P^3^ was 20,487 N (Table 3). This magnitude was substantially greater than P^3^ experienced under the load case with all muscles active, and with constraints at the tooth and both jaw joints. The reaction force was anteriorly directed, indicating that the tooth would impose posteriorly-directed shearing force on the food.

### Bite force magnitudes compared with other mammals

**Fig 4:**
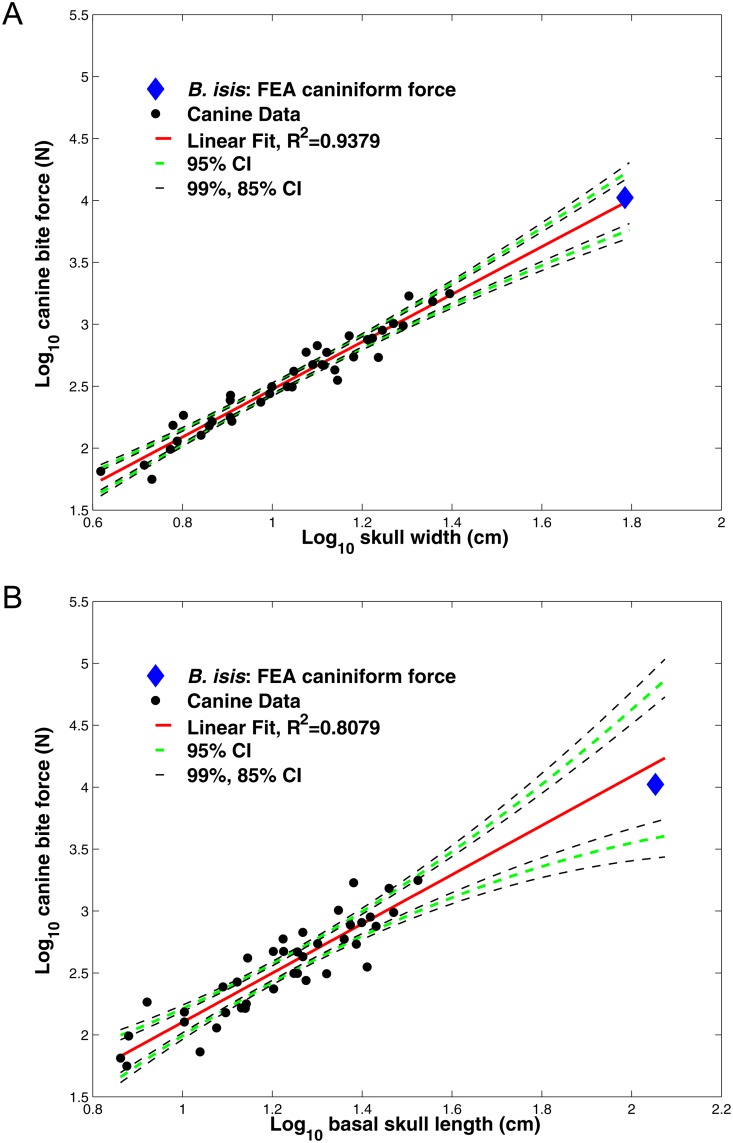
Regressions of canine bite force against skull width and basal skull length in carnivorous mammals. (A) Regressions of log10-transformed canine bite forces against log10 skull width and (B) regressions of log10-transformed canine bite forces against log10 basal skull length, in carnivorous mammals. Canine bite force values are compiled from Wroe et al. (2005), and listed in Table 3. Isometric specific tension is 30 N/cm^2^. *Basilosaurus isis* (blue diamond) has a slightly greater FEA-estimated bite force at the caniniform I^2^ (at the same anterior position as the canine in most carnivorous mammals) than expected from the skull width equation (A), but lower force than expected from its skull length (B). Note that these values fall well within 95% confidence intervals for the entire sample, suggesting that *B*. *isis* did not have exceptionally high or low bite force compared with that expected of a mammal with its skull dimensions.

Fig. 4 shows regressions for mammalian terrestrial carnivores from data in Table 4, and Table 5 compares regression estimates of *Basilosaurus* bite force with results of FE analyses. FE-estimated forces for *B*. *isis* are close to those expected from its skull width at both the caniniform I^2^ and at its more posterior, actual canine (9.6% and 15.7% greater, respectively, than the expected 9,614 N). However, *B*. *isis*’s FEA-estimated canine-position force is 32.5% lower than expected for its skull length and its canine force is 28.8% lower. The FEA-derived forces for *B*. *isis* are within the 95% (and even 85%) confidence intervals of the regression (Fig. 4), whereas residuals for many mammals in the initial regressions fall outside these bounds. The simulated forces for *B*. *isis* thus are not exceptionally high or low compared with predicted values. The skull width/bite force regression is tighter (R^2^ = 0.938) than the regression for length/force (R^2^ = 0.808).

**Table 4:** Regression of canine bite force against basal skull length and skull width.

	log-10 BSL (cm)	log-10 SW(cm)	log-10 CB (N)
*Alopex lagopus*	1.142	0.906	2.250
*Canis alpinus*	1.248	1.033	2.497
*Canis aureus*	1.131	0.910	2.217
*Canis lupus dingo*	1.256	0.999	2.496
*Canis lupus hallstromi*	1.203	0.974	2.371
*Lycaon pictus*	1.268	1.139	2.631
*Vulpes vulpes*	1.140	0.866	2.215
*Urocyon cineroargentus*	1.076	0.788	2.057
*Canis latrans*	1.275	0.994	2.439
*Canis lupus lupus*	1.360	1.121	2.773
*Canis dirus*	1.418	1.245	2.951
*Ursus americanus*	1.387	1.236	2.733
*Ursus arctos*	1.431	1.212	2.876
*Ursus thibetanus*	1.321	1.044	2.494
*Meles meles*	1.090	0.906	2.387
*Gennetta tigrinus*	1.039	0.715	1.863
*Crocuta crocuta*	1.374	1.223	2.888
*Hyaena hyaena*	1.301	1.181	2.736
*Proteles cristatus*	1.096	0.859	2.179
*Panthera onca*	1.347	1.270	3.006
*Panthera tigris*	1.460	1.357	3.183
*Acinonyx jubatus*	1.202	1.090	2.674
*Felis yagouaroundi*	1.004	0.841	2.104
*Lynx rufus*	0.880	0.773	1.991
*Felis concolor*	1.225	1.111	2.674
*Felis sylvestris*	0.876	0.732	1.748
*Neofelis nebulosa*	1.224	1.075	2.775
*Panthera leo*	1.524	1.395	3.247
*Panthera pardus*	1.256	1.115	2.669
*Smilodon fatalis*	1.470	1.291	2.989
*Dasyurus maculatus*	1.004	0.779	2.185
*Dasyurus viverrinus*	0.862	0.618	1.813
*Sarcophilus harrisii*	1.145	1.048	2.621
*Nimbacinus dicksoni*	1.122	0.907	2.427
*Thylacinus cynocephalus*	1.399	1.171	2.907
*Priscileo roskellyae*	0.921	0.802	2.265
*Wakaleo vanderleurei*	1.268	1.100	2.828
*Thylacoleo carnifex*	1.381	1.304	3.228
*Thylacosmilus atrox*	1.411	1.145	2.548
***Basilosaurus isis***	2.053	1.785	-

Data for regressions of log-10 canine bite force (CB) against log-10 of basal skull length (BSL) and skull width (SW) in carnivorous mammals from Wroe et al. [26]. Forces assume a specific tension of 30 N/cm^2^. Log-10 BSL and SW measurements are given for *Basilosaurus isis*.

**Table 5:** Regression-predicted and FE-reaction “canine” bite forces in *Basilosaurus isis*.

	Caniniform I^2^		Canine	
**Predicted forces (N)**	log 10	Absolute	log 10	Absolute
Skull length regression	4.194	**15617**		
Skull width regression	3.983	**9614**		
2D lever model		**9219**		**12655**
**FEA forces (N)**				
ST = 30 N/cm^2^	4.023	**10536**	4.046	**11122**
ST = 37 N/cm^2^	4.114	12995	4.137	13716

Predicted forces are from regressions of force at the canine position (I^2^ in *Basilosaurus*) against skull measurements in carnivorous mammals. The FEA results are bite reaction forces at I^2^ and, for comparison, at the true canine. Results in boldface all assume 30 N/cm^2^ specific tension of jaw muscles, as in Wroe et al. (2005); note similarity between the finite element and skull width-predicted forces.

Based on the lever model, reaction forces at I^2^ and the canine were 9,219 N and 12,655 N with the assumption of 30 N/cm^2^ specific muscle tension, compared with the 10,536 N and 11,122 N values from the FEA model. These results suggest that the methods give adequately similar results for gross comparisons of forces derived from FEA and extrapolated from regression of lever models. However, using a single vertical resultant force (as with this lever model) gave unpredictably different results (+/– 10%) compared with our asymmetrical FE model with 3D component and reaction forces.

## Discussion

This study of *Basilosaurus isis* reveals the utility of simple FE representations of skeletal geometry (plate and solid-filled models) for obtaining reaction forces. As photogrammetry and other surface-modelling methods proliferate [59], [60], [61], [62], FE models based on this surface data alone can be useful for estimating reaction forces associated with biting and locomotion [58]. However, stress magnitudes and distribution require traditional, continuously advancing CT- based volumetric models [63], [64], [65]. Plate [41], [66], [67], structurally abstracted [68], and cavity-filled models are recommended primarily for broad comparisons of stress distribution. Encouragingly, both types of simplified models are biologically informative for estimating absolute reaction forces, as shown here for *Basilosaurus* biting (Fig. 2).

### Ability of *Basilosaurus isis* to crush bone

With all muscles active, unilateral estimated tooth reaction forces for this specimen of *Basilosaurus isis*, at a realistic 37 N/cm^2^ [29], were about 16,400 N at P^3^ regardless of model plate thickness and von Mises stress (Fig. 2). Including the medial pterygoid muscles would likely increase this force. This magnitude greatly exceeds forces necessary to crush or comminute bone with blunt-edged or rounded conical teeth [69], [70], [71], [72]. Captive spotted hyenas exert about 3,500 N [72] and wild hyenas perhaps double this value [73]. Indentation of a *Triceratops* ilium by a *Tyrannosaurus rex* tooth required 6,410 N [69], and *Basilosaurus* forces at P^3^ exceed that study’s extrapolated value (13,400 N) for the *T*. *rex*’s posterior teeth [69]. Finite element-modeled anterior bite forces in *B*. *isis* (10,536–13,716 N, depending on tooth position and specific tension: Table 5) are also sufficient to indent bone.

Under the orthal retraction load case, anteriorly-directed food reaction forces of about 20,000 N (Table 3), on the small area of tooth-food contact, would likely exceed the ultimate (breaking) shear stress of bone tissue. These high reaction forces are consistent with the heavy macroscopic tooth wear seen in WH 74, as well as the suggested consumption of large hard objects such as mammal bones [23]. Because muscle force of a unilateral bite would induce a moment about the tooth and retract the contralateral side, we suggest that *Basilosaurus isis* would moderate the muscle forces it applied during such behaviors. The von Mises stress at the tooth under the orthal retraction load case (>200 MPa; Table 3) greatly exceeds sheer stress of bone or dentine. This suggests deficiency of the plate element model, but also that the animal might moderate forces to below the maximum theoretical value. Reaction forces would be lower with more teeth in contact with the food, and still exceed shear strength of prey tissues. Traditional FEA with a continuous-solid model is necessary to test the plate models’ results for stress magnitude and distribution (Figs. 2 and 3); we predict grossly similar distribution but lower magnitudes of von Mises stress, because the specimen’s cranial bone is often very thick.

### Implications of bite force in *Basilosaurus isis* for predation and scavenging

Fahlke [24] considered *Basilosaurus isis* as a likely predator that included juvenile *Dorudon atrox* in its diet. Distribution of bite marks indicates that *B*. *isis* bit *D*. *atrox* calves across the head from a lateral position, and sometimes adjusted prey in the mouth prior to a more powerful bite that penetrated the bone. High bite forces that break bone enable efficient carcass processing, whether an animal kills prey or is scavenging [71], [74].

We interpret high bite forces in *Basilosaurus isis* as indicating capability for habitual predation, rather than exclusive scavenging. Fahlke [24] did not rule out scavenging for *B*. *isis*, and scavenging occurs among large marine carnivores. Shark bite marks on fossils [75], [76] and forensics on modern animals indicate that sharks both scavenge and prey upon marine mammals [77], and did so upon mosasaurs during the Cretaceous [75]. However, pure scavenging among endotherms is known only in energy-efficient soaring birds [78], [79], including turkey vultures (*Cathartes aura*), which have weak bites and pedal grips compared with other carnivorous birds (ES, pers. obs.). In contrast with scavenging birds, the greatest bite forces known are from extant carnivores observed killing prey (crocodilians and white sharks: [80]), or extinct forms that broke bones of live prey which escaped, and whose bones healed (e.g. *Tyrannosaurus rex*: [81], [82], and giant sharks: [76], [80]). *B*. *isis* had comparable bite forces to these predators (Table 6). Healed bite marks in *Dorudon* could confirm predaceous habits for *B*. *isis*, but the cause(s) of the few healed injuries that are known in *Dorudon* [10] could not be identified unequivocally.

**Table 6:** *Basilosaurus isis* bite force estimates (N) compared with other long-skulled carnivores, including skull length and method.

	Method	Skull length (m)	Posterior F 37 N/cm^2^|max 30N/cm^2^	Anterior F 37 N/cm^2^|max 30N/cm^2^
***Basilosaurus isis*** jaws articulated	FEA	1.13	16461|20020	12994–13717| 16026–16918
***Basilosaurus isis*** orthal retraction	FEA	1.13	20487|24844 (ant. shear)	n/a
*Kronosaurus queenslandicus* ^*1*^	FEA	1.8	27716	15169
*Pliosaurus kevani* ^*2*^	FEA	2	27865–48278	11865–20884
*Crocodylus porosus* ^*3*^ (4.59 m)	force transducer	0.65	16414	11216
*Crocodylus porosus* ^*3*^ (6.7 m)	extrapolated transducer	-	27531–34424	-
*Deinosuchus* ^*3*^ *riograndensis*	extrapolated transducer	1.3–2	102803	-
*Tyrannosaurus rex* ^*4*^	dynamics	1.3	35640–57158	18065–31086
*Tyrannosaurus rex* ^*5*^	indentation	-	13400	6410
*Tyrannosaurus rex* ^*4*,*6*^	extrapolated dynamics	1.3	105732	53593
*Dunkleosteus terreli* ^*7*^	dynamics	0.8	7495	5625
*Carcharodon carcharias* ^*8*^	FEA	-	18216	9320
*Carcharodon / Carcharocles megalodon* ^*8*^	extrapolated FEA		108514	55522

Ranges are cited when available. Tooth reaction forces of *B*. *isis* are given for 37 N/cm^2^ muscle specific tensions, and the maxima (max) calculated from common 1.5X underestimates of mammalian bite force at a specific tension of 30 N/cm^2^. Anterior bite forces for *B*. *isis* vary with tooth position. Note that *B*. *isis* bite forces are lower than in large-headed reptiles (especially crocodylians and *Tyrannosaurus rex*). However, considering its shorter skull, forces in *B*. *isis* are comparable to estimates for the marine pliosaurs *Kronosaurus* and *Pliosaurus*. Sources: ^1^[36], ^2^[37], ^3^[30], ^4^[28], ^5^[69], ^6^[52], ^7^[89], ^8^[80].

### *Basilosaurus isis* bite force compared with other large-headed carnivores and smaller mammals

The great diversity of methods for estimating bite forces (Table 6) warrants caution when comparing our results for *Basilosaurus isis* with forces for other animals. Imperfections of our method likely underestimate bite force in *B*. *isis*. These include omission of the medial pterygoid for its primary role in jaw lateral movements in mammals, and posteriorly-originating muscles that cause only jaw reaction forces, rather than taking advantage of the moment arm of the coronoid process. Underestimating masseter cross-section would also underestimate the total bite force. Applying the dynamics-based methods of Bates and Falkingham [28] to *B*. *isis* would allow surer comparisons of *B*. *isis* forces with their results for large reptiles, particularly for impact bite force.

Despite these caveats, bite force estimates for *Basilosaurus isis* appear to be comparable to those of very large white sharks [80], although relatively lower than those of some large-headed reptilian predators of similar skull length (Table 6). Reptiles have a laterally unconstrained, multi-aponeurosis m. pterygoideus posterior/ventralis that loops around the lower jaw, and pennate temporal muscles with greater forces per ACSA than the 30–37 N/cm^2^ specific tension (*ST*) values for mammals [25], [29]. (When isometric *ST* for *Tyrannosaurus* [28] is scaled to *ST* of the tuatara *Sphenodon* [52], the tyrannosaur’s posterior bite forces reach the 100,000 N values estimated through structural mechanics [83], and calculated for giant crocodilians [30].) Forces remain lower in *B*. *isis* even with greater estimates of specific tension in mammalian jaw muscles. Thomason found that the dry skull method can underestimate mammalian bite forces at 30 N/cm^2^ by 1.3–1.5 [25]. Scaling up to these values to assume 39–45 N/cm^2^ of specific tension, maximum estimates for mandible elevation in *B*. *isis* (load case 1) would therefore be 17,350–20,020 N using our method. These reaction forces are still lower than estimated for the largest *Crocodylus porosus* [30] (Table 6).

Despite relatively lower forces than in crocodylians and one dinosaur, our bite force estimates for *Basilosaurus isis* are similar to estimates for marine pliosaurs *Kronosaurus queenslandicus* and *Pliosaurus kevani* [36], [37], when considering the longer skulls of these reptiles. Assuming that muscle force is proportional to the square of linear increases in size, a *B*. *isis* with a 2 m skull would be expected to have a bite force of about 50,000 N: (2m/1.13m)^2^ = 3.13; 3.13 x 16,451 N = 51,523 N. This value is in the range of 48,000 N estimated for *Pliosaurus kevani* with a 2 m skull, and may suggest similar trade-offs of hydrodynamics and bite force in these large marine carnivores [36], [37].

More directly instructive for *Basilosaurus* bite force and feeding style, the ratios of anterior/posterior bite forces that we estimate for *B*. *isis* (Table 6) are 15–22% greater than the ratio recorded in *Crocodylus porosus* [30]. This suggests that *B*. *isis* applied relatively greater anterior bite forces than crocodilians for the same posterior forces (about 16,000 N in both *B*. *isis* and a 4.6 m *C*. *porosus*), and maintained effective bone-crushing abilities at all tooth positions. High anterior bite force in *B*. *isis* would also enable it to capture and hold large prey with its widely-spaced anterior teeth, possibly prior to processing it, a predation technique Uhen [10] suggested for *Dorudon atrox*.

Our conservative modeled bite force estimates for *Basilosaurus isis* are the largest known for any mammal, and are much greater than in bone-breakers like spotted hyenas. With comparable specific tensions of 30 N/cm^2^, the anterior caniniform (I^2^) bite force of *B*. *isis* (10,536 N: Table 5) is over twice the canine force estimated for the giant ursid *Agriotherium africanum* (4,566 N: [84]). *Basilosaurus isis* had somewhat greater estimated bite force than expected from regressions of bite force versus skull width in carnivorous mammals (Table 3, Fig. 4), and lower force than expected for its skull length. Its elongated rostrum probably accounts for the lower-than-expected values from the length regression, and its relatively narrow braincase may have given *B*. *isis* relatively more muscle cross-sectional area and force than in mammals with broader braincases compared with their overall skull width. However, FEA and lever model results are not dramatically different from those expected from regressions, falling well within confidence intervals. Comparisons with terrestrial mammals such as the huge *Megistotherium* [85] and mesonychid *Andrewsarchus* [86], and cetartiodactyls of the land-water transition, will place *B*. *isis* bite force in productive comparative biomechanical and evolutionary context.

For example, comparing *Basilosaurus* with other whales will be informative about predatory ecomorphology, both at the time of their Eocene radiation and in adaptation to certain prey. *Basilosaurus* was certainly specialized among the aquatic archaeocetes, as is implied not only by its destructive tooth wear but also by its unusually elongate vertebrae and consequently serpentine body shape (cf. [87], [88]). From the evolutionary aspect, it would therefore also be interesting to conduct a bite force analysis for the more generalized dorudontine basilosaurids. Finally, our understanding of the ecological role of *B*. *isis* would benefit from comparisons of its bite force with forces estimated for modern aquatic mammals that have a similar range of prey items, e.g., the killer whale (*Orcinus orca*).

## Summary and Conclusions

Bite force of the middle-to-late Eocene archaeocete *Basilosaurus isis* from Egypt was estimated using FEA modeling. Bite reaction forces varied negligibly with FE element formulation. Resulting maximum bite forces for *B*. *isis* are conservative, yet are the highest ever estimated or recorded for a mammal, and are comparable with or only moderately lower than many bite forces recorded or estimated for large reptiles (e.g. *Crocodylus porosus*, pliosaurs, and *Tyrannosaurus rex*) and white sharks. Bone crushing was definitely possible for *B*. *isis*, potentially even when it was using its anterior teeth, and estimated bite force in the anterior teeth is relatively higher than in reptiles, suggesting *B*. *isis* was capable of manipulating large prey using its canines and incisors. Very high bite forces at P^3^ and farther anteriorly are consistent with *B*. *isis* being an active predator rather than a scavenger. *B*. *isis* was probably a specialist among archaeocetes, and comparing its bite force with those of other extinct and extant cetartiodactyls will place our results into evolutionary and ecological context more comprehensively.

## Supporting Information

S1 Dataset Autodesk Multiphysics finite element model of *Basilosaurus isis*. (FEM)

S1 Text Use of plate elements for vertebrate FEA. Elaborates on the use of plate elements for vertebrate FEA.
(DOCX)
