# Supplementary material for: Bone-Breaking Bite Force of Basilosaurus isis (Mammalia, Cetacea) from the Late Eocene of Egypt Estimated by Finite Element Analysis
Source: PLoS One. 2015 Feb 25;10(2):e0118380. doi: 10.1371/journal.pone.0118380 (PMC4340796; doi:10.1371/journal.pone.0118380)
Supplement: S1 Text — Elaborates on the use of plate elements for vertebrate FEA. (DOCX) [file pone.0118380.s002.docx]

**Supporting Information for**

Bone-Breaking Bite Force of *Basilosaurus isis* (Mammalia, Cetacea) from the Late Eocene of Egypt Estimated by Finite Element Analysis

Eric Snively, Julia M. Fahlke, Robert C. Welsh (PLoS ONE)

**Use of plate elements for vertebrate FEA**

Plate elements are rarely used in FE simulations of vertebrate function, because skeletal structures are more complex than simple walled structures. However, plate elements are useful even with meshes consisting of mainly tetrahedral or hexahedral brick elements. For example, including plate elements can introduce varying material properties to otherwise uniform models. Plate elements assigned to the surface of a bone model’s volumetric mesh can be given the thickness and properties of its cortical bone [1], leaving cancellous bone properties for the interior mesh.

Compared with brick elements, plate elements are more efficient for a given number of degrees of freedom (x, y, and z displacement/node), in part because some formulations do not require calculation of transverse normal and shear stresses [2]. This enables quicker solutions and lower computing resources for a given number of elements. Conversely, this efficiency facilitates use of large meshes, which give greater confidence that a model captures peak stress and strain values [3], [4]. In FEA convergence studies, successively-greater mesh resolutions asymptotically approach the results from the highest-resolution mesh [3], [4]. Smaller meshes with negligibly different peak stress relative to the largest will be more computationally economical. Ideally a model has enough elements for sufficient accuracy, yet is economical enough to enable many simulations for a given amount of computing time. Because plate element models solve rapidly at high resolution, they allow rapid analyses of many loading regimes and testing of related hypotheses.

A third benefit of plate elements is that bending is included their formulation [2]. Plate elements are therefore more accurate than solid internal elements for stress and strain derived from bending loads. At least with simple structures, results from plate-element models more closely approach analytical solutions from beam theory (e.g. biological examples of [5] and [6]), or experimentally measured displacements and strains [3].

Although plate element models enable rapid FE exploration of hypothesized loads, they have cautionary disadvantages compared with volumetric meshes. Plate element models are unlikely to capture internal stresses and strains of complex structures as well as a high-resolution internal mesh ([3], [4]), despite their mathematical efficiencies. For complex cross-sections (unless their thickness is continuously varied), plate elements will not necessarily approach analytically calculated stresses [5] better than volumetric FEA.

Results from this study indicate that plate element FE analyses give precise reaction forces regardless of element thickness, and are therefore useful for bite force studies. They enable FE modelling of bite force from surface models (laser-scanned or photogrammetric: [7]), from CT scans of permineralized fossils with noise artifacts that obscure internal structure, and from morphologically accurate, and CT-based computer aided design reconstructions [8]. However, stress magnitudes and distribution from plate element FEA are best treated as hypotheses to be tested with volumetric meshes.

**Supporting Information References**

1. Eichenseer PH, Sybert DR, Cotton JR **(**2011) A finite element analysis of sacroiliac joint ligaments in response to different loading conditions. Spine 36: E1446-1452.
2. Hughes TJR (2000) The finite element method: linear static and dynamic finite element analysis. Mineola: Dover Publications Inc. 662 p.
3. Bright JA, Rayfield EJ (2011) The response of cranial biomechanical finite element models to variations in mesh density. Anat Rec 294: 610–620.
4. Tseng ZJ, Mcnitt-Gray JL, Flashner H, Wang X, Enciso R (2011) Model sensitivity and use of the comparative finite element method in mammalian jaw mechanics: mandible performance in the Gray Wolf. PLoS ONE 6(4): e19171.
5. Porro LB, Holliday CM, Anapol F, Ontiveros LC, Ontiveros LT, Ross CF (2011) Free body analysis, beam mechanics and finite element modelling of the mandible of *Alligator mississippiensis*. J Morphol 272: 910–937.
6. Walmsley CW, Smits PD, Quayle MR, McCurry MR, Richards H, Oldfield C, Wroe S, Clausen PD, McHenry CR (2013) Why the long face? The mechanics of mandibular symphysis proportions in crocodiles. PLoS ONE 8(1): e53873.
7. Falkingham PL (2012) Acquisition of high resolution 3D models using free, open-source, photogrammetric software. Palaeontol Electronica 15(1) 1T:15p.
8. McHenry CR, Clausen PD, Daniel WJT, Meers MB, Pendharkar A (2006) Biomechanics of the rostrum in crocodilians: a comparative analysis using finite-element modeling. Anat Rec Part A 288A: 827-849.
